# Editorial to “The incidence and risk factors of atrial high‐rate episodes in patients with a dual‐chamber pacemaker”

**DOI:** 10.1002/joa3.13153

**Published:** 2024-09-23

**Authors:** Kenji Yodogawa

**Affiliations:** ^1^ Department of Cardiovascular Medicine Nippon Medical School Tokyo Japan

Atrial fibrillation (AF) is the most common chronic supraventricular arrhythmia, which is associated with thromboembolic complications and heart failure. The early detection of AF is important to avoid those events, but difficult particularly in patients with asymptomatic AF.

Recently, continuous rhythm monitoring with cardiac implantable electronic devices has been used to diagnose brief episodes of arrhythmia, including paroxysmal AF, which are recorded as atrial high‐rate episodes (AHRE). A recent systematic review and meta‐analysis demonstrated that the overall AHRE incidence ratio was estimated to be 17.56 cases per 100 person‐years. Although AHREs were not associated with a statistically significant increased mortality risk, patients with documented AHREs were 4.45 times more likely to develop clinical AF, and were associated with a 1.90‐fold increased stroke risk.^1^ Thus, early detection of AHRE is also crucial to improve prognosis in patients with cardiac implantable electronic devices.

Pastori et al. evaluated 496 consecutive patients with cardiac implantable electronic devices. They found that AHRE were recorded in 173 patients, and multivariable Cox regression analysis showed that age, prior AF, white cell count, and high C reactive protein were independently associated with AHRE. However, clinical scores using age, left atrial size, renal function, ejection fraction, metabolic syndrome, and cardiomyopathy were nonsignificantly associated with AHRE. Similar results were obtained for CHADS2 and CHA2DS2VASc score.^2^

P‐wave dispersion (PWD) is an ECG parameter and predictor of AF, which is defined as the difference between the maximum and the minimum P‐wave durations detected on the body surface 12‐lead ECG. Nishinarita et al. reported that PWD was an independent predictor of new‐onset AHRE. They showed a greater incidence of sick sinus syndrome and longer PWD were apparent in the AHRE than non‐AHRE group. In logistic regression analysis, receiver‐operating characteristic curve analysis (area under the curve 0.90; *p* < .001) suggested the best cutoff value for PWD was 48 mm (sensitivity 73.8%, specificity 77.9%).^3^

Recently, the four‐dimensional automatic LA quantitative analysis (4D Auto LAQ) technology in real‐time three‐dimensional echocardiography (RT‐3DE) has been developed. Using this technology, Wang et al. investigated predicting factors for AHRE. Left atrial contraction longitudinal strain (LASct) obtained by the technology, body surface area (BSA), and LA end‐systolic volume (LAESV) were influencing factors for AHRE. Multivariate analysis revealed that LASct was an independent risk factor for the AHRE.^4^

In this study, the authors examined the prevalence and risk factors associated with the occurrence of AHRE in patients with a dual‐chamber pacemaker. Left ventricular global longitudinal strain (GLS‐LV) was measured by speckle tracking echocardiography. They found that the prevalence of AHRE after 6 months follow‐up was 30.34%, and history of antiarrhythmic drug use, history of paroxysmal supraventricular tachycardia, percentage of premature atrial contraction on 24‐h Holter electrocardiogram before implantation, and GLS‐LV are the independent predictors for AHRE.^5^

Their findings may provide useful information for the management of patients with intracardiac devices. However, as they described, the role of anticoagulation therapy to patients with AHRE detected by intracardiac ECG remains controversial. Further studies are ongoing to evaluate the benefit of oral anticoagulants in patients with AHRE.

Taken together, various factors were reported to be associated with AHRE so far. A large‐scale, randomized control study is warranted to reveal independent risk factors for AHRE.

## CONFLICT OF INTEREST STATEMENT

Authors declare no conflict of interests for this article.
